# OpenWorm: overview and recent advances in integrative biological simulation of *Caenorhabditis elegans*

**DOI:** 10.1098/rstb.2017.0382

**Published:** 2018-09-10

**Authors:** Gopal P. Sarma, Chee Wai Lee, Tom Portegys, Vahid Ghayoomie, Travis Jacobs, Bradly Alicea, Matteo Cantarelli, Michael Currie, Richard C. Gerkin, Shane Gingell, Padraig Gleeson, Richard Gordon, Ramin M. Hasani, Giovanni Idili, Sergey Khayrulin, David Lung, Andrey Palyanov, Mark Watts, Stephen D. Larson

**Affiliations:** 1 School of Medicine, Emory University, Atlanta, GA, USA; 2 The OpenWorm Foundation, New York, NY, USA; 3 Ernst and Young LLP, New York, NY, USA; 4 Laboratory of Systems Biology and Bioinformatics, University of Tehran, Tehran, Iran; 5 Melonport AG, Zug, Switzerland; 6 Orthogonal Research, Champaign, IL, USA; 7 Fling Inc., Bangkok, Thailand; 8 Laboratory of Complex Systems Simulation, A.P. Ershov Institute of Informatics Systems, Novosibirsk, Russia; 9 Laboratory of Structural Bioinformatics and Molecular Modeling, Novosibirsk State University, Novosibirsk, Russia; 10 Department of Neuroscience, Physiology and Pharmacology, University College London, London, UK; 11 Embryogenesis Center, Gulf Specimen Marine Laboratory, Panacea, FL, USA; 12 C.S. Mott Center for Human Growth and Development, Department of Obstetrics and Gynecology, Wayne State University, Detroit, MI, USA; 13 Cyber-Physical Systems, Technische Universität Wien, Wien, Austria; 14 Raytheon Company, Waltham, MA, USA; 15 Out of the BOTS, Inc., Queensland, Australia; 16 School of Life Sciences, Arizona State University, Tempe, AZ, USA

**Keywords:** computational neuroscience, bioinformatics, software engineering, computational physiology, biological simulation, *Caenorhabditis elegans*

## Abstract

The adoption of powerful software tools and computational methods from the software industry by the scientific research community has resulted in a renewed interest in integrative, large-scale biological simulations. These typically involve the development of computational platforms to combine diverse, process-specific models into a coherent whole. The OpenWorm Foundation is an independent research organization working towards an integrative simulation of the nematode *Caenorhabditis elegans*, with the aim of providing a powerful new tool to understand how the organism's behaviour arises from its fundamental biology. In this perspective, we give an overview of the history and philosophy of OpenWorm, descriptions of the constituent sub-projects and corresponding open-science management practices, and discuss current achievements of the project and future directions.

This article is part of a discussion meeting issue ‘Connectome to behaviour: modelling *C. elegans* at cellular resolution’.

## 1. Introduction

In 2011, the OpenWorm project was launched with the mission of building the world's first detailed biophysical simulation of the nematode *Caenorhabditis elegans* [1,2]. In addition to the ambitious scientific goals, a unique aspect of the project is the fully open science, distributed research framework in which the work would take place. In this article, we look at the past, present and future of OpenWorm. What has it achieved in the period since its foundation, what are the important next steps and what can others learn from this experience?

A unifying principle underpinning OpenWorm is the application of an engineering approach to the challenge of managing biological complexity [3]. Modern software engineering has given us the tools to keep track of the hundreds of thousands of details of which complex physical systems are composed. The synergy between human and machine in computer-assisted modelling can allow for deeper reasoning than either a human or computer alone. In industrial manufacturing, for example, advances in engineering software have enabled materials simulations that allow mechanical engineers to test many different mechanisms *in silico* before the manufacturing process [4]. While the fields of computational biology and computational neuroscience have made significant advances over their multi-decade history, simulations have only had a limited impact on the biological thinking process when compared with other disciplines in the physical and engineering sciences [5].

What level of complexity should our model aim for? Our perspective is the following: *an integrative model need not incorporate any more detail than the individual models the research community has already produced*. In other words, we take a holistic approach in which individual models, which may operate at multiple scales, are thoughtfully integrated into a unified computational platform, providing a global view of the entire organism. As we will discuss below, the lowest level of biological detail that the OpenWorm project incorporates is that of ion channel models which underpin membrane potential dynamics. Examples such as the whole cell model of Karr *et al.* [6] demonstrate that this kind of ‘holistic biology’ can lead to valuable insights into underlying biological function [6]. The purpose of this work is integrative and allows us to extract even greater value from the knowledge the scientific community has already produced. Nowhere is the need for models that encompass multiple scales more evident than in the hermaphrodite nematode's network of 302 neurons, where simple crawling and swimming behaviours remain unexplained [7]. Despite decades of effort, we struggle to describe how individual neurons give rise to such diverse organismal behaviour. Our belief is that a computational platform in which an organism's behaviour arises from lower-level biological models will come to play a significant role in advancing the field.

In the field of *C. elegans* biology, there has been significant effort to collect comprehensive anatomical and other structural data about the nervous system, ranging from the electrical and synaptic connectome [8], to cholinergic and GABAergic neurons [9,10], to the extrasynaptic connectome of neuropeptides [11]. The purpose of generating these ‘map’-like datasets is to communicate the relationships between biological entities. Unfortunately, the complexity of such datasets places severe limitations on their intelligibility. This problem is not unrelated to modern genomics, where the many-tangled webs of relationships between hundreds of thousands of genes and gene products demand computational tools to assist in their understanding. It is with this complexity in mind that the OpenWorm project has taken upon itself to integrate the disparate and heterogeneous physiological maps and related datasets generated by the *C. elegans* community into a coherent software framework. Efforts such as PyOpenWorm (described below) are one such example in OpenWorm where publicly available data are assembled into a graph database and Python application programming interface, enabling users to query multiple datasets about *C. elegans* neuronal structure. By creating an open, shared repository and query tool for these data, the fruits of collective labour become integrated into a shared structure that amplifies the impact of the entire community's research output. Moreover, the need to arrive at a global view of relevant datasets has allowed us to identify key areas where new data should be collected, potentially taking advantage of novel experimental apparatus such as robotic patch-clamp set-ups. Ultimately, we expect that unified platforms for data integration will dovetail with other contemporary efforts in the life sciences to increase the robustness and exchangeability of datasets and models [12–14].

In the scientific community, assembling datasets solely for the purpose of consolidation has often led to the emergence of multiple, redundant standards. In the OpenWorm project, our fundamental aim is to curate datasets and mathematical models in a manner that facilitates dynamic simulations of biological function*.* Theoretical biophysicists have produced a rich literature of quantitative models of *C. elegans* physiology, ranging from membrane potential dynamics, to neuromuscular coupling, to the fluid dynamics of body movement. Integrating these individual models into a global, composite simulation creates an additional check on the underlying datasets themselves. In addition, the simulation enables the construction of complex hypotheses which researchers can further investigate through theoretical or experimental means [5].

In deciding on the level of biological detail we wish to incorporate, we have agreed upon an approach that incorporates biomechanics as a critical component of understanding the nematode in the context of its environment [15]. In addition to the biological implications, maintaining biological realism may have implications well beyond understanding *C. elegans*. Indeed, researchers in the artificial intelligence community have posited that sensorimotor feedback may play a role in allowing future AI systems to learn from experience more efficiently than current data-hungry systems based on deep learning [16]. As such, we have unified a biomechanical model of *C. elegans*, Sibernetic [17,18], that incorporates interactions with a fluid or gel environment, with a modelling infrastructure for complex neuronal networks, c302 [19].

Our ultimate vision for OpenWorm is to provide a computational platform that allows for simulations to become seamlessly integrated into biological thought. Rather than replacing existing theoretical or experimental methods, our vision is to take advantage of the powerful tools of modern software engineering to maximally enable the research community and leverage long-standing intellectual traditions and biological insights [5]. We can imagine a number of possible applications for such a platform. Because we have complete control over all details of the simulation, we can effortlessly create knockouts, where, for example, all synaptic connections to or from a specific cell can be removed. We can simulate known mutants that have ion channels with different properties and observe their behaviour. We can simulate the effects of drugs by modelling their impact on ion channels, potentially paving the way to using simulations as a way to generate hypotheses for new uses of existing pharmacological agents and for discovering new ones. If successful in the *C. elegans* community, we would hope this approach could assist in the understanding of other organisms in biology. In the remainder of this paper, we describe progress in the open resources we have produced, their uses and features, and future directions for the project.

## 2. Material and methods

### (a) Software infrastructure for simulating *C. elegans*

#### (i) OpenWorm simulation stack

**Figure 1.**
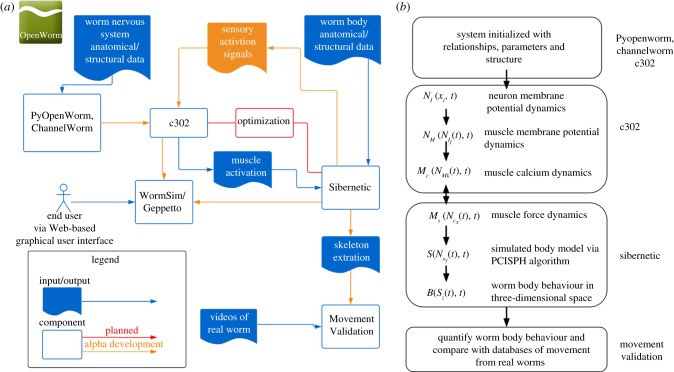
Overview of OpenWorm simulation stack. (*a*) A component diagram describing the relationships between inputs and outputs of sub-projects within OpenWorm. (*b*) A highly simplified schematic view of the system of equations executed in the combined c302/Sibernetic system.

OpenWorm is organized into a number of sub-projects, several of which are described in more detail in this issue. In this section, we will give a condensed overview of the core of the platform. A ‘simulation stack’ refers to the set of integrated software tools that are used to run a simulation. It is called a ‘stack’ because each tool can be thought of as existing at a certain level in a hierarchy of abstraction and information flow. For instance, at the lowest level, we have ion channel models and connectomes. At the next level, we have models of neuromuscular coupling. And finally, the output generated by the connectome can be fed into a simulation of the body movement and environment. While each element of this simulation stack could form the basis for an independent research project, our aim is to use best practices from the software industry to integrate these tools into a single software framework. Figure 1 shows the different components of the OpenWorm simulation stack and their relationships.

Figure 1*a* shows a breakdown of the contents of the OpenWorm simulation stack described as components of software. Inputs and outputs to the software components are depicted with arrows showing how they relate to the core modules. For the PyOpenWorm and ChannelWorm software projects, inputs include anatomical and structural data from the worm's nervous system and knowledge about ion channels, respectively. These data are fed into the c302 software component, which constructs systems of equations that are used to simulate the membrane dynamics of the nervous system at multiple levels of detail ranging from simple integrate-and-fire neurons to multi-compartment neuronal models [19]. The outputs of the c302 simulation include muscle activation signals which form the inputs to the Sibernetic system. We are planning on incorporating feedback from Sibernetic to c302 that represents sensory signals generated from the worm body's posture as well as interactions with the environment. Additionally, Sibernetic takes as an input structural and biophysical data about the worm. The output from Sibernetic, the outline of the worm's body as it bends and moves over time, can be fed into the movement validation software system, where comparisons with videos of real worms are used to validate the global model's biological validity. These two systems will be incorporated into a web-based graphical user interface framework that provides a visual interface to the end user via WormSim/Geppetto [20]. An optimization block in the diagram indicates where the free parameters in the models can be filled in by tuning model parameters of single neurons to match experimental data [21,22].

In figure 1*b*, we show a simplified schematic that breaks down the integrated c302/Sibernetic system into mathematical components. The system is initialized with relationships, parameters and structure derived from databases that have been populated with information about *C. elegans* physiology. In the current version of the simulation, we begin with neuron membrane potential dynamics (N_I_) that are set manually. From those dynamics, the electrical activities of the body wall muscle cells (N_M_), i.e., those muscles receiving direct synaptic input from neurons, are calculated. This activation also results in dynamical changes in the muscles' internal calcium concentration (M_c_). These first components of the simulation are carried out in the c302 framework, which executes a NeuroML-based model in the NEURON simulation engine [19]. The calcium dynamics of the muscle cells calculated by c302 (M_c_) are passed into Sibernetic as activation signals. These activation signals are converted into forces that cause activated muscle cells lining the body model to contract (M_s_). The combination of the contraction states of all the muscles leads to the state of the simulated body model as a whole (S), calculated via the predictive–corrective incompressible smoothed particle hydrodynamics (PCISPH) algorithm for modelling fluids. The aggregate of all of the particles that make up the simulated body model is the behaviour of the simulated worm over time (B). This can then be compared against the movement of real worms once brought into a comparable format [23]. While there is currently only a uni-directional flow of information from c302 to Sibernetic via muscle activation signals, we are developing a reverse step where forces on the skin of the worm body model lead to activation signals of sensory neurons.

#### (ii) PyOpenWorm

Biological data are often weakly structured and heterogeneous, which creates fundamental problems for computational platforms that rely on these data. In addition, discrepancies that are frequently seen between database formats and term definitions create even further difficulties for end users. The challenges in making use of biological data are common across all subfields of computational biology, with *C. elegans* being no exception. PyOpenWorm (https://github.com/openworm/pyopenworm) is a Python package intended to simplify access to a range of structured data on *C. elegans* anatomy and physiology. It is a data access layer for *C. elegans* information, where users can query data across multiple scales of the worm's biology. The heterogeneous nature of *C. elegans* biology requires that different underlying technologies be used to store different types of data. For instance, an RDF semantic graph representation is useful for representing neuronal structural properties such as ion channel expression and the density and type of neurotransmitter receptors, whereas a NeuroML representation is most appropriate for storing model morphology and simulation parameters [24]. PyOpenWorm solves the problem of abstracting away the underlying technologies, so the user can query the system in a manner that is intuitive for researchers who are already familiar with the worm's biology. The resulting data can be used directly or as part of a multistage software pipeline. The software project is implemented in the Python programming language and the code is available on GitHub along with the other sub-projects of OpenWorm.

Data from reliable external sources, most often published journal articles, are collected into a single directory in the PyOpenWorm repository. These datasets can take the form of structured spreadsheet files or even other relational databases. For quality control, we only consider data that have an original source associated with them. Currently, data are collected from the literature and other secondary sources, such as WormBase [25] and WormAtlas [26]. When a user or program connects with PyOpenWorm's database, they have access to all of the data through a simple Python library. Table 1 lists current data sources that are part of PyOpenWorm.

**Table 1.** Data sources incorporated into PyOpenWorm (https://pyopenworm.readthedocs.io/en/latest/data_sources.html).

data type	source
neurons and muscles	
names	WormBase; Harris *et al*. [25]
neuron types, cell descriptions, lineage names, neurotransmitters, neuropeptides, receptors, innexins	WormAtlas; Altun *et al*. [27]
monoamine secretors and receptors, neuropeptide secretors and receptors	Bentley *et al*. [11]
connectome	
neuron to neuron and neuron to muscle chemical synapses and gap junctions	personal communication by S. Cook (original data set available at http://bit.ly/2MGiv9K ); White *et al*. [8]

Although PyOpenWorm's primary current use cases are for storing static data and models, its fundamental architecture anticipates future needs once members of the research community begin to make use of the OpenWorm tool stack as part of their daily research. In particular, ongoing development of PyOpenWorm is aimed at ensuring that the system can store metadata and simulation results, so that this output can subsequently be interrogated and analysed as part of the research process.

#### (iii) ChannelWorm

As we discussed above, ion channels represent the most granular level of biological detail that the OpenWorm simulation incorporates. Ion channels are pore-forming proteins, found in the membranes of all cells. They are responsible for many known cellular functions including shaping action potentials and gating the flow of ions across the cell membrane. Remarkably, most nematode ion channels are conserved across vertebrate species [28]. Because of their widespread relevance for biology, many electrophysiological experiments have been focused on ion channels and transporter functional genomics in *C. elegans* [29–34]. Although much of this work is experimental, computational work has also been directed at integrating ion channel models into larger-scale simulations [35–40]. One such example (outside *C. elegans* biology) is the Blue Brain Project, which recently unveiled a detailed simulation of a rat cortical micro-column [41], taking advantage of an extensive repository of curated data and models of ion channels [42].

We have chosen ion channel models as the most granular level of detail with which to simulate the nematode for several reasons. For instance, insights into drug development would not be possible without an understanding of the action of the major neurotransmitter species on Na^+^, K^+^ and Ca^2+^ currents. Fortunately, incorporating ion channel models is a tractable approach and there is no need to limit ourselves to simulations of more abstract neurons. Moreover, the specific dynamics of ion channels themselves are key components of the models of neuromuscular coupling that we use. And as we argued above, biomechanics is a central component of our scientific roadmap.

As part of the OpenWorm project, we created ChannelWorm (https://github.com/openworm/channelworm and https://chopen.herokuapp.com) in order to (i) integrate and structure data related to ion channels in *C. elegans*, (ii) digitize and curate electrophysiological data from publications, (iii) develop application programming interfaces for accessing these data and (iv) build ion channel models based on experimental data. As the project has progressed, we have found ourselves in the unique position of attempting to develop a global view of the current state of *C. elegans* ion channel modelling. One of the major lessons we have learned is that patch-clamp data are only available for a small minority of ion channels expressed in the nematode. Consequently, a significant undertaking is to build Hodgkin–Huxley models for ion channels that lack these data based on homologous channel types from other organisms. After a manual curation process in which contributors digitize electrophysiological plots, kinetic parameters are derived from these data using genetic algorithms [22,43,44] and related techniques such as particle swarm optimization. Ultimately, these ion channel models are translated into the NeuroML markup language [45,46], which allows for consistent representation of neuronal biophysics, anatomy and network architecture for use in subsequent computational simulations.

#### (iv) Software testing and model validation

As a software project, OpenWorm shares many commonalities with any large-scale software engineering endeavour in industry. *Unit testing* is a key element of modern software engineering which uses semi-automated checklists to ensure the correctness of software. For instance, a company developing a word processor might have a test that verifies whenever the mouse clicks a specific region in the upper left hand of the screen, the ‘File’ menu opens and not the ‘Edit’ menu. Likewise, other tests might verify that files can be appropriately written to disk or that connectivity with printers and other network devices is working. From its inception, OpenWorm has incorporated best practices from the software industry, including unit testing, across all of the diverse sub-projects, especially PyOpenWorm [47]. Examples of unit tests used by OpenWorm include verifying that entries can be added to and removed from the PyOpenWorm database, that every biological fact such as ion channel parameters have associated PubMed identifiers and that functions implement error handling correctly.

As a scientific research project that incorporates dynamic models, another class of tests crucial to our effort are *model validation tests.* In contrast to simple unit tests, which verify that a discrete piece of code has the correct behaviour, model validation tests verify that the output of an entire dynamic model corresponds to known behaviour from the academic literature. For instance, alongside the ion channel curation and parameter extraction tasks in ChannelWorm, a parallel effort is aimed at implementing validation tests for each of these models using the Python library SciUnit [48]. The validation process uses curated datasets of ion channel behaviour to instantiate analogous statistical tests that a researcher would use when developing such a model. By incorporating this process into the software development workflow, we can ensure that developers and researchers are alerted if any of the models at any level of abstraction are not in correspondence with known behaviour determined by experimentalists [47,49,50].

### (b) Outreach, education and sister projects

#### (i) Web-based visualization of OpenWorm models

We recognize that many motivated and talented citizen scientists are not experienced in software engineering and data science. Consequently, to make the OpenWorm model as accessible as possible, we have worked to create simple and intuitive applications that can be used for exploratory purposes and which can serve as a fun and compelling entry point to the project. Initial work to accomplish this was the development of the WormSim (http://wormsim.org) prototype. WormSim was launched via a successful Kickstarter campaign in 2014, but this has been superseded by more advanced approaches to visualizing these models. Recent developments with the Geppetto platform (http://geppetto.org) [20] for multi-scale biological simulation, which was the underlying platform for WormSim, have enabled users to visualize the *C. elegans* connectome within the body of the worm itself, and visualize and explore changing dynamics in the connectome to see the effect on swimming and crawling. This version of the visualization is currently being incorporated into the OpenWorm simulation stack above in order to allow users to examine intermediate levels of the simulation (see [20], this issue, for visual examples.)

#### (ii) Robotics

Because the scientific vision of OpenWorm places a key emphasis on biomechanics, we have multiple outlets for how the virtual nervous system simulation interfaces with the world. One is through a fully virtual body embedded in a virtual physical environment. Another is for the nervous system simulation to interact directly with a robotic body, a platform that provides a unique educational opportunity for newcomers to engage with the project.

**Figure 2.**
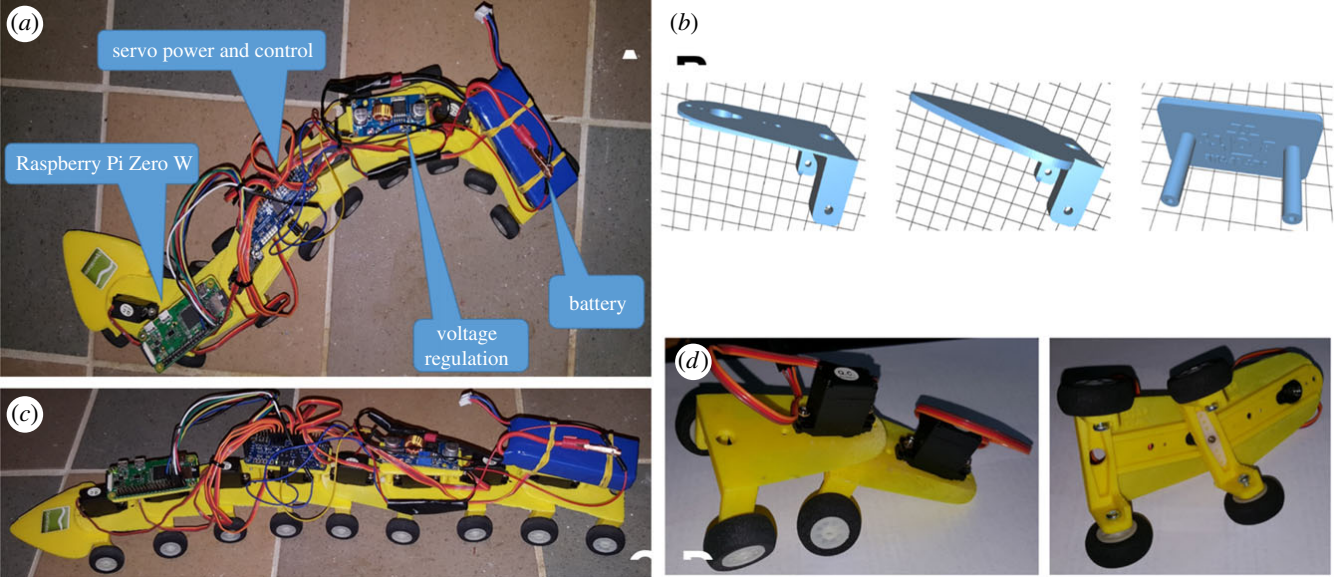
(*a*) Top view of the robot. (*b*) Three-dimensional-printed body parts. (*c*) Side view of the robot. (*d*) Segment sub-assembly.

Figure 2*a*,*c* shows a top and side view of a prototype OpenWorm robot (https://github.com/openworm/robots) with major components denoted. The robot consists of nine articulated segments, each segment mounted on a pair of wheels. Locomotion is achieved, as it is in *C. elegans*, by moving in a snake-like manner that relies on surface friction. The wheels are not powered and exist solely to provide a suitable contact surface with the ground. Each segment is a three-dimensional-printed component (figure 2*b*) that articulates with its neighbours via servos (figure 2*d*). The electronic components, consisting of the Raspberry Pi Zero microprocessor with wireless communication capabilities, are mounted on platforms fastened to several of the segments. A pulse-width modulation board distributes power and controls signals from the Raspberry Pi Zero to the servos. Each servo is capable of maintaining a specified angular position that translates to inter-segment angular positioning.

Figure 2*b* shows the designs for the 3 three-dimensional-printed parts. These parts are specified in a common .stl file format that is editable and portable to most three-dimensional printers. On the left is the segment part. In the centre is the head that is envisaged to be mounted with sensors for food foraging and touch. On the right is one of the platforms for mounting the electronic components. Figure 2*d* shows how the segments are articulated. A servo is mounted on the front top of the segment with its geared shaft extending into an aperture in the next forward segment. An arm secured to the gear allows for gear motion to drive angular movement between segments.

Like WormSim, the robotics sub-project of OpenWorm is a key element of our education and outreach efforts. The accessibility and low cost of electronics microprocessors like the Raspberry Pi make this an attractive and compelling introduction to the project for students of all ages, which exposes them to bleeding edge concepts at the intersection of software and robotics. Ongoing work in the robotics sub-project is aimed at incorporating models for food foraging and touch response, developing a new system-on-a-board processor that will also perform power and control distribution, utilizing laser-cut segments and providing a programming interface via Jupyter notebooks.

#### (iii) DevoWorm

Much of what we have described above pertains to simulations of the adult nematode. A complementary goal for computational research, with direct relevance for many members of the *C. elegans* experimental community, is to simulate embryogenesis and development in *C. elegans*. Given the knowledge of the embryonic cell lineage in *C. elegans* [51], one of the goals of DevoWorm, an ongoing sister project to OpenWorm with a parallel set of approaches (https://github.com/devoworm), is to apply a similar modelling technique of transforming datasets into computable forms and evolving their progression over time using mathematical models of biophysical developmental processes. It is currently divided into three loosely knit sub-projects: Developmental Dynamics, Cybernetics and Digital Morphogenesis, and Reproduction and Developmental Plasticity.

Developmental Dynamics currently involves using secondary data collected from embryos [52,53] along with bioinformatic and data science techniques to answer questions regarding the process of early embryogenesis and the timing of later morphogenesis. Cybernetics and Digital Morphogenesis has involved using cellular automata [54] or finite-element approaches [55] to model physical interactions during embryogenesis and morphogenesis. DevoWorm has also explored the use of cybernetic models and concepts to better understand the general process of embryogenesis [56]. Reproduction and Developmental Plasticity involves an evolutionary developmental biology approach [57] to understand *C. elegans* more generally. DevoWorm's existing datasets and papers include a focus on larval development and life-history processes. Taken together, these focus areas are beginning to draw additional interest into simulated embryogenesis and morphogenesis of *C. elegans*.

### (c) Community management support

#### (i) Distributed scientific collaboration

A citizen science consortium with over 90 contributors^1^ from 16 different countries and no central source of funding, OpenWorm has been an organizational experiment in coordinating a distributed, international research effort with a highly fluid base of contributors. Freely available software tools have played a key role in project management and coordination. The focal point of much of our work is the diverse functionality of the GitHub platform [58], which allows us to use sophisticated, industrial-scale management tools for versioning the OpenWorm codebase as well as data from our university-based research partners.

Other platforms such as the Google Docs platform with spreadsheets, drawings, slides and forms have also been critical for the creation and distribution of shared materials. Teleconferencing systems like Google Hangouts have enabled building trust, camaraderie and working relationships among contributors living in many time zones across the globe. Google Calendar has been invaluable for scheduling, as has the Doodle poll tool for coordinating meeting times. The functionality of the Slack chat platform has played a crucial role in managing the many asynchronous conversations related to software development and the scientific roadmap. Given the volume of high-quality tools such as Amazon Web Services, Docker, Slack and many others that are available for use in the modern era of software engineering, the challenges we faced in the initial stages of building the organization often amounted to making the right choices about which tools to use on the basis of the cohesiveness of their relationships with one another. Consequently, the integration points between these different systems have been one of the concrete deliverables of OpenWorm for other organizations interested in distributed community management.

Because of its open source and volunteer-based nature, timelines for task completion are often fluid. Coordinating the project requires the discovery of synergy among collaborators based on individual interests and research goals. Managing the project requires creating the potential for others to contribute and build, connecting that potential to the right individuals at the right time and ensuring that there is sufficient flexibility in the high-level vision so that the project can make progress even if all directions are not advancing at a given moment.

The ‘long memory’ of online resources is helpful in this regard. Issues that are captured in GitHub may sit inactive for months before the right person comes along who has the skill set and motivation to solve them. Consequently, the tolerance of contributors to uncertainty is an important component of working well within an open community. We have taken inspiration from the open source programming movement that follows a similar philosophy. In open source software development, new volunteers are encouraged to take personal responsibility and leadership for creating new directions that excite them. A unique aspect of research and development in an open community is the rate at which volunteers enter the project eager to learn and to contribute their time and energy to a shared effort that is larger than any one individual [59].

#### (ii) Mentorship and training through badges

Open-science projects face a very different set of management challenges when compared with university or industry-based research initiatives. In particular, mechanisms are needed to assist new contributors to develop relevant technical skills and build familiarity with the project. To facilitate this process, OpenWorm has taken advantage of a free service called BadgeList, which allows for the creation of digital ‘micro-credentials’ certifying that an individual is able to complete a focused set of tasks (http://badgelist.com/openworm). Upon successfully learning and answering a set of test questions, a user can earn a badge, indicating that they have acquired a specific skill set. Example badges currently used by the project include basic and advanced GitHub/version control, Hodgkin–Huxley equation basics and literature mining. The collection of badges has been growing over the past several years, and many new contributors have found the system to be a valuable entry point to the project.

#### (iii) Volunteer composition and project leadership

We have been fortunate that OpenWorm has attracted an incredibly diverse set of volunteers with respect to nationality and intellectual background. As we mentioned above, we have over 90 volunteers from 16 different countries who have made substantive contributions to the project. Moreover, the contributors have come from a variety of academic backgrounds, including theoretical and experimental biology, physics and computer science, to name just a few. In addition to several core members who are tenure-track faculty at major universities, many of our volunteers are professional software engineers. One area where we are keen to make more progress in is the gender diversity within the project. We have recognized this as a priority and have advertised on social media our active commitment to providing a safe and welcome space for all individuals. We welcome any input on how we might go about achieving a more equitable gender balance.

We are frequently asked about project leadership, decision-making and conflict resolution. Like many open source projects, our list of contributors has a long tail, with a few core contributors assuming leadership roles and many others making periodic, smaller contributions [60,61]. To date, we have had no formal process for assigning roles, and we have found that experienced and enthusiastic volunteers often establish themselves as leaders without any prompting. Subgroups dedicated to topics ranging from engineering, to basic science, to community outreach organize via dedicated channels on Slack, and new volunteers have the opportunity to contribute to whichever efforts resonate with them the most. We have actively worked to ensure a culture where open deliberation takes place with all contributors receiving a voice. With the formal incorporation of the OpenWorm Foundation as an independent, non-profit research organization, we have formed an official scientific advisory board that is responsible for establishing the scientific direction of the effort. Thus far, we have found that input from the scientific advisory board has organically filtered into the project in an effective manner. As the project grows, we may consider formalizing the roles of full-time staff and primary collaborators.

## 3. Recent progress

What progress has the OpenWorm project made since the publication of our first overview paper [1]? The number of contributors has grown substantially and the codebase has sufficiently matured that new volunteers can join and begin to contribute by tackling open issues on GitHub. Building on several years of experience managing an open-science project, as well as our collective experience building software in the commercial and academic setting, we have refined many of our management practices to better serve the needs of a fluidly shifting base of contributors. The badge system described above has been used by several dozen new members, and we have been holding weekly ‘office hours’ on Slack where senior contributors are available to answer questions.

With regard to more traditional academic metrics, the special issue in which this article appears will include the publication of several new articles featuring foundational modelling, simulation, data management and data presentation technologies developed as a result of OpenWorm-led collaborations [19,20,23,48]. Before this special issue, we have published a handful of papers on several different facets of the project in a spectrum of journals focused on the computational biological sciences. We have been involved with multiple academic conferences and have built university-based collaborations with six different research laboratories in four countries. Equally as important, we have formally been incorporated as an independent non-profit research organization, the OpenWorm Foundation. This foundation has allowed us to assemble an accomplished scientific advisory board that is helping to guide us through this critical infrastructure-building phase of the project.

To address a query frequently asked of the project: ‘when can we turn the simulation on?’, the simple answer is that there is already prototype code to do this, available online at GitHub (http://github.com/openworm/openworm). The *C. elegans* connectome contained in c302 is able to drive body movement in the Sibernetic platform for fluid dynamic simulations. However, the level of detail that we have incorporated to date is inadequate for biological research. A key remaining component is to complete the curation and parameter extraction of Hodgkin–Huxley models for ion channels to produce realistic dynamics in neurons and muscles. Once this task is complete, we expect that the platform will incorporate a sufficiently granular level of detail to be of interest to researchers in the field.

Table 2 summarizes our accomplishments.

**Table 2.** Recent achievements of the project.

result type	accomplishments
scientific communication	‘Connectome to Behavior’ conference at The Royal Society, London, UK, 2018
	Workshop at Neural Information Processing Systems (NIPS), Los Angeles, USA, 2017
	Genetics Society of America 22nd International *C. elegans* Conference Workshop, Los Angeles, USA, 2017
community efforts	Office hours—a weekly meeting on Slack open to anyone where senior contributors are available to answer questions about the project
	Badges—16 badges providing ‘micro-credentials’ for key skills necessary for contributing to OpenWorm. Fifty-one badges have been earned by contributors since the beginning of the project.
	Journal clubs—YouTube-based series reviewing scientific papers relevant to modelling *C. elegans*
	Mailing list—1600 subscribed members
distributed project management tools	8 612 788 lines of code in 51 different programming languages spanning 63 sub-projects (repositories) in GitHub
	Slack workspace has 171 members, 43 weekly active users across 27 public channels
	Twitter account has 3000 followers, average monthly impressions: 25 000, maximum impressions: 46 000 in January 2018.
organizational	OpenWorm Foundation incorporated as a 501c(3) in the USA in 2016. Formed a formal board of directors and a scientific advisory board (http://openworm.org/people.html).
academic collaborations	University College London, London, UK
	Imperial College, London, UK
	Arizona State University, AZ, USA
	A.P. Ershov Institute of Informatics Systems, Novosibirsk, Russia
	TU Wien, Vienna, Austria
	Emory University School of Medicine, GA, USA
publications	Four new publications from OW contributors and collaborators in submission for special issue at *Phil. Trans. Royal Soc.*
	Previous articles listed at: http://openworm.org/publications.html

## 4. Discussion

**Figure 3.**
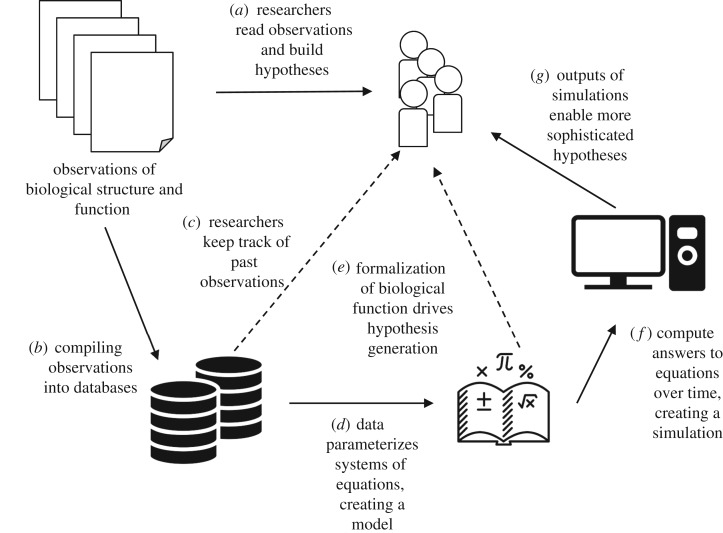
Schematic view of the scientific value of modelling and simulation in biology, as applied in OpenWorm.

By organizing the research output of an entire community into a shared structure, integrative simulations have the potential to advance biological thinking significantly. Rather than being replacements for existing theoretical or experimental techniques, these composite simulations should be viewed as powerful tools to augment the thought process and technical toolbox of scientists. Figure 3 summarizes how integrative simulations can be an organic part of the research process. The same observations that researchers use to form mental models and hypotheses are first organized into databases such as PyOpenWorm and ChannelWorm (arrow *a*). Researchers benefit from these databases directly, for example, by having on-demand access to useful facts about *C. elegans* physiology (arrows *b* and *c*). Subsequently, these datasets are formalized into mathematical models, a process that is itself intrinsically valuable to researchers as part of hypothesis generation (arrow *e*). Most significant are the final steps of this sequence, in which the datasets and mathematical models are integrated into a larger, composite simulation. By studying the outputs of simulations, analogous to the outputs of experiments, researchers are able to augment their intuition and mental models about biological function in ways that would not be possible through experimentation alone (arrows *f* and *g*).

While these agendas are in their nascent stages, many of the key components of the OpenWorm simulation platform will only need to be built once and can then be re-used community-wide in day-to-day research. At the OpenWorm Foundation, we are assembling the necessary technical and organizational infrastructure to build the world's first integrative biological simulation of the nematode *C. elegans*. We hope that subsequent efforts will benefit from our experience, and, in the future, we hope to see the vision of integrative biological simulations extend to many other model organisms and have a widespread scientific impact.

### (a) Future directions

Looking forward, there are two thrusts for the project: a primarily scientific one, and the second, a primarily engineering or tool-building phase.

The tap withdrawal circuit is a well-studied experimental protocol we are currently investigating that has focused our transition from infrastructure development to actively using the platform for scientific research [62,63]. Simulating this behaviour will require closing the loop between sensation, motor output and environmental activity. In addition, the nervous system model must be able to transform an external input into a switch of behaviour from crawling forwards to backwards. As a prerequisite, we must also implement a version of forward and backward locomotion based on the activity of motor neurons driving the muscles of the model. To ensure the correctness of such a model, we are incorporating SciUnit-based model validation tests, which allow us to constrain the simulation to match experimental data at different scales and modalities. A critical component of this research direction will be efficient optimization algorithms to help fill in data gaps of free parameters that are currently unknown within the biological community. Once a working prototype of tap withdrawal is completed, we can look at perturbing the model in ways that are consistent with mutations known to have an impact on neuronal or other cellular activity. This will be a valuable test of the ability of the OpenWorm integrated model to capture essential dynamics despite significant biological variation.

Our engineering aim at present is to reach a steady state where the fundamental infrastructure of OpenWorm has stabilized and can be used for scientific research. Active ongoing infrastructural development in the project includes expanding the functionality of PyOpenWorm to store metadata and provenance of simulations, using this framework to build a database of simulation results, completing the ion channel curation and parameter extraction tasks in ChannelWorm, building an automated system for identifying new publications on *C. elegans* relevant for OpenWorm and expanding the automated framework for verifying the correctness of curated scientific models, to name just a few. More information about making a contribution is available on our website and via our volunteer contribution form (http://bit.ly/OpenWormVolunteer).

## Endnote

1 Contributors are defined as any individual who has updated code in our open source repositories or contributed organizationally or scientifically. See http://openworm.org/people.html.

## Data accessibility

All data and code associated with OpenWorm is available through our GitHub repository at https://github.com/OpenWorm.

## References

[1] Szigeti B et al. 2014 OpenWorm: an open-science approach to modeling *Caenorhabditis elegans*. Front Comput. Neurosci. 8, 137 (10.3389/fncom.2014.00137) 25404913 PMC4217485

[2] Gleeson P, Cantarelli M, Currie M, Hokanson J, Idili G, Khayrulin S, Palyanov A, Szigeti B, Larson S 2015 The OpenWorm Project: currently available resources and future plans. BMC Neurosci. 16, P141 (10.1186/1471-2202-16-S1-P141)

[3] Harel D 2004 A grand challenge for computing: towards full reactive modeling of a multi-cellular animal. In Verification, model checking, and abstract interpretation (eds Steffen B, Levi G), pp. 323–324. Berlin, Germany: Springer

[4] Avgoustinov N 2007 Modelling in mechanical engineering and mechatronics: towards autonomous intelligent software models. London: Springer See https://market.android.com/details?id=book-dLVrtwAACAAJ.

[5] Sarma GP, Faundez V 2017 Integrative biological simulation praxis: considerations from physics, philosophy, and data/model curation practices. Cell Logist. 7, e1392400 (10.1080/21592799.2017.1392400) 29296511 PMC5739097

[6] Karr JR, Sanghvi JC, Jacobs JM, Macklin DN, Covert MW 2012 A whole cell model of mycoplasma genitalium elucidates mechanisms of bacterial replication. Biophys. J. 102, 731a (10.1016/j.bpj.2011.11.3967)22385843

[7] Gjorgjieva J, Biron D, Haspel G 2014 Neurobiology of *Caenorhabditis elegans* locomotion: where do we stand? Bioscience 64, 476–486. (10.1093/biosci/biu058) 26955070 PMC4776678

[8] White JG, Southgate E, Thomson JN, Brenner S 1986 The structure of the nervous system of the nematode *Caenorhabditis elegans*. Phil. Trans. R. Soc. Lond. B 314, 1–340. (10.1098/rstb.1986.0056) 22462104

[9] Pereira L et al. 2015 A cellular and regulatory map of the cholinergic nervous system of *C. elegans*. eLife 4, 299 (10.7554/eLife.12432) PMC4769160 26705699

[10] Gendrel M, Atlas EG, Hobert O 2016 A cellular and regulatory map of the GABAergic nervous system of *C. elegans*. eLife 5, 1395 (10.7554/eLife.17686) PMC5065314 27740909

[11] Bentley B, Branicky R, Barnes CL, Chew YL, Yemini E, Bullmore ET, Vértes PE, Schafer WR 2016 The multilayer connectome of *Caenorhabditis elegans*. PLoS Comput. Biol. 12, e1005283 (10.1371/journal.pcbi.1005283) 27984591 PMC5215746

[12] Le Novere N et al. 2006 BioModels database: a free, centralized database of curated, published, quantitative kinetic models of biochemical and cellular systems. Nucleic Acids Res. 34, D689–D691. (10.1093/nar/gkj092) 16381960 PMC1347454

[13] Ascoli GA, Donohue DE, Halavi M 2007 NeuroMorpho.Org: a central resource for neuronal morphologies. J. Neurosci. 27, 9247–9251. (10.1523/JNEUROSCI.2055-07.2007) 17728438 PMC6673130

[14] Milyaev N, Osumi-Sutherland D, Reeve S, Burton N, Baldock RA, Armstrong JD 2012 The Virtual Fly Brain browser and query interface. Bioinformatics 28, 411–415. (10.1093/bioinformatics/btr677) 22180411

[15] Tytell ED, Holmes P, Cohen AH 2011 Spikes alone do not behavior make: why neuroscience needs biomechanics. Curr. Opin. Neurobiol. 21, 816–822. (10.1016/j.conb.2011.05.017) 21683575 PMC3183174

[16] Hay N, Stark M, Schlegel A, Wendelken C, Park D, Purdy E, Silver T, Phoenix DS, George D 2018 Behavior is everything—towards representing concepts with sensorimotor contingencies. In 32nd AAAI Conf. Artif. Intell., New Orleans, LA, 2–7 February 2018, pp. 1861–1870. See https://www.aaai.org/ocs/index.php/AAAI/AAAI18/paper/view/16413. Palo Alto, CA: AAAI Press.

[17] Palyanov A, Khayrulin S, Larson SD 2016 Application of smoothed particle hydrodynamics to modeling mechanisms of biological tissue. Adv. Eng. Softw. 98, 1–11. (10.1016/j.advengsoft.2016.03.002)

[18] Palyanov A, Khayrulin S, Larson SD 2018 Three-dimensional simulation of the *Caenorhabditis elegans* body and muscle cells in liquid and gel environments for behavioural analysis. Phil. Trans. R. Soc. B 373, 20170376 (10.1098/rstb.2017.0376) 30201840 PMC6158221

[19] Gleeson P, Lung D, Grosu R, Hasani R, Larson SD 2018 c302: a multi-scale framework for modelling the nervous system of *Caenorhabditis elegans*. Phil. Trans. R. Soc. B 373, 20170379 (10.1098/rstb.2017.0379) 30201842 PMC6158223

[20] Cantarelli M, Marin B, Quintana A, Earnshaw M, Court R, Gleeson P, Dura-Bernal S, Silver RA, Idili G 2018 Geppetto: a reusable modular open platform for exploring neuroscience data and models. Phil. Trans. R. Soc. B 373, 20170380 (10.1098/rstb.2017.0380) 30201843 PMC6158222

[21] Masoli S, Rizza MF, Sgritta M, Van Geit W, Schürmann F, D'Angelo E 2017 Single neuron optimization as a basis for accurate biophysical modeling: the case of cerebellar granule cells. Front. Cell Neurosci. 11, 71 (10.3389/fncel.2017.00071) 28360841 PMC5350144

[22] Gurkiewicz M, Korngreen A 2005 A numerical approach to ion channel modelling using whole-cell voltage-clamp recordings and a genetic algorithm. PLoS Comput. Biol. 3, e169 (10.1371/journal.pcbi.0030169.eor) PMC1963494 17784781

[23] Javer A, Ripoll-Sánchez L, Brown AEX 2018 Powerful and interpretable behavioural features for quantitative phenotyping of *Caenorhabditis elegans*. Phil. Trans. R. Soc. B 373, 20170375 (10.1098/rstb.2017.0375) 30201839 PMC6158219

[24] de Bono B, Hunter P 2012 Integrating knowledge representation and quantitative modelling in physiology. Biotechnol. J. 7, 958–972. (10.1002/biot.201100304) 22887885

[25] Harris TW, Antoshechkin I, Bieri T, Blasiar D 2009 WormBase: a comprehensive resource for nematode research. Nucleic Acids 38, D463–D467. (10.1093/nar/gkp952) PMC2808986 19910365

[26] Altun ZF, Hall DH 2002 WormAtlas. See http://www.wormatlas.org.

[27] Altun ZF, Herndon LA, Wolkow CA, Crocker C, Lints R, Hall DH (eds). 2002–2018 WormAtlas. See http://www.wormatlas.org.

[28] Bargmann CI 1998 Neurobiology of the *Caenorhabditis elegans* genome. Science 282, 2028–2033. (10.1126/science.282.5396.2028) 9851919

[29] Goodman MB, Ernstrom GG, Chelur DS, O'Hagan R, Yao CA, Chalfie M 2002 MEC-2 regulates *C. elegans* DEG/ENaC channels needed for mechanosensation. Nature 415, 1039–1042. (10.1038/4151039a) 11875573

[30] Strange K 2003 From genes to integrative physiology: ion channel and transporter biology in *Caenorhabditis elegans*. Physiol. Rev. 83, 377–415. (10.1152/physrev.00025.2002) 12663863

[31] Salkoff L, Wei AD, Baban B, Butler A, Fawcett G, Ferreira G, Santi CM 2005 Potassium channels in *C. elegans*. WormBook 1–15. (10.1895/wormbook.1.42.1) PMC4781360 18050399

[32] Hobert O 2005 Specification of the nervous system. WormBook 1–19. 10.1895/wormbook.1.12.1 PMC4781215 18050401

[33] Bianchi L, Driscoll M 2006 Heterologous expression of *C. elegans* ion channels in *Xenopus* oocytes. WormBook 1–16. 10.1895/wormbook.1.117.1 PMC4781024 18050441

[34] Liu P, Chen B, Wang Z-W 2014 SLO-2 potassium channel is an important regulator of neurotransmitter release in *Caenorhabditis elegans*. Nat. Commun. 5, 5155 (10.1038/ncomms6155) 25300429 PMC4197135

[35] Hodgkin AL, Huxley AF 1952 A quantitative description of membrane current and its application to conduction and excitation in nerve. J. Physiol. 117, 500–544. (10.1113/jphysiol.1952.sp004764) 12991237 PMC1392413

[36] Willms AR, Baro DJ, Harris-Warrick RM, Guckenheimer J 1999 An improved parameter estimation method for Hodgkin-Huxley models. J. Comput. Neurosci. 6, 145–168. (10.1023/A:1008880518515) 10333160

[37] Boyle JH, Cohen N 2008 *Caenorhabditis elegans* body wall muscles are simple actuators. Biosystems 94, 170–181. (10.1016/j.biosystems.2008.05.025) 18619514

[38] O'Leary T, Williams AH, Franci A, Marder E 2014 Cell types, network homeostasis, and pathological compensation from a biologically plausible ion channel expression model. Neuron 82, 809–821. (10.1016/j.neuron.2014.04.002) 24853940 PMC4109293

[39] Mirzakhalili E, Epureanu B, Gourgou E 2017 A mathematical and computational model of the calcium dynamics in *Caenorhabditis elegans* ASH sensory neuron. PLoS ONE 13, e0201302 (10.1371/journal.pone.0201302) PMC6062085 30048509

[40] Kuramochi M, Doi M 2017 A computational model based on multi-regional calcium imaging represents the spatio-temporal dynamics in a *Caenorhabditis elegans* sensory neuron. PLoS ONE 12, e0168415 (10.1371/journal.pone.0168415) 28072834 PMC5224993

[41] Markram H et al. 2015 Reconstruction and simulation of neocortical microcircuitry. Cell 163, 456–492. (10.1016/j.cell.2015.09.029) 26451489

[42] Ranjan R, Khazen G, Gambazzi L, Ramaswamy S, Hill SL, Schürmann F, Markram H 2011 Channelpedia: an integrative and interactive database for ion channels. Front. Neuroinform. 5, 36 (10.3389/fninf.2011.00036) 22232598 PMC3248699

[43] Milescu LS, Akk G, Sachs F 2005 Maximum likelihood estimation of ion channel kinetics from macroscopic currents. Biophys. J. 88, 2494–2515. (10.1529/biophysj.104.053256) 15681642 PMC1305347

[44] Wang W, Xiao F, Zeng X, Yao J, Yuchi M, Ding J 2012 Optimal estimation of ion-channel kinetics from macroscopic currents. PLoS ONE 7, e35208 (10.1371/journal.pone.0035208) 22536358 PMC3335051

[45] Gleeson P et al. 2010 NeuroML: a language for describing data driven models of neurons and networks with a high degree of biological detail. PLoS Comput. Biol. 6, e1000815 (10.1371/journal.pcbi.1000815) 20585541 PMC2887454

[46] Cannon RC, Gleeson P, Crook S, Ganapathy G, Marin B, Piasini E, Silver RA 2014 LEMS: a language for expressing complex biological models in concise and hierarchical form and its use in underpinning NeuroML 2. Front. Neuroinform. 8, 79 (10.3389/fninf.2014.00079) 25309419 PMC4174883

[47] Sarma GP, Jacobs TW, Watts MD, Ghayoomie SV, Larson SD, Gerkin RC 2016 Unit testing, model validation, and biological simulation. F1000Res. 5, 1946 (10.12688/f1000research.9315.1) 27635225 PMC5007758

[48] Gerkin RC, Jarvis RJ, Crook SM 2018 Towards systematic, data-driven validation of a collaborative, multi-scale model of *Caenorhabditis elegans*. Phil. Trans. R. Soc. B 373, 20170381 (10.1098/rstb.2017.0381) 30201844 PMC6158230

[49] Gerkin RC, Omar C 2013 NeuroUnit: validation tests for neuroscience models. Front. Neuroinform. (10.3389/conf.fninf.2013.09.00013)

[50] Omar C, Aldrich J, Gerkin RC 2014 Collaborative infrastructure for test-driven scientific model validation In Companion Proc. of the 36th Int. Conf. Software Engineering, pp. 524–527. ACM.

[51] Sulston JE 1983 Neuronal cell lineages in the nematode *Caenorhabditis elegans*. Cold Spring Harb. Symp. Quant. Biol. 48, 443–452. (10.1101/SQB.1983.048.01.049) 6586366

[52] Santella A et al 2015 WormGUIDES: an interactive single cell developmental atlas and tool for collaborative multidimensional data exploration. BMC Bioinformatics 16, 189 (10.1186/s12859-015-0627-8) 26051157 PMC4459063

[53] Wang E, Santella A, Wang Z, Wang D, Bao Z 2017 Visualization of 3-dimensional vectors in a dynamic embryonic system—WormGUIDES. J. Comput. Commun. 5, 70–79. (10.4236/jcc.2017.512008)

[54] Portegys T, Pascualy G, Gordon R, McGrew SP, Alicea BJ 2017 Morphozoic, cellular automata with nested neighborhoods as a metamorphic representation of morphogenesis. In Multi-agent-based simulations applied to biological and environmental systems. (ed. Adamatti DF), pp. 44–80. Hershey, PA, USA: IGI Global.

[55] Izaguirre JA et al. 2004 CompuCell, a multi-model framework for simulation of morphogenesis. Bioinformatics 20, 1129–1137. (10.1093/bioinformatics/bth050) 14764549

[56] Gordon R, Stone R 2017 Cybernetic embryo. In Biocommunication (eds Gordon R, Seckbach J), pp. 111–164. London, UK: World Scientific.

[57] Carroll SB 2005 Endless forms most beautiful: the new science of Evo devo and the making of the animal kingdom. WW Norton & Company See https://market.android.com/details?id=book-CnnGKjzw3xMC.

[58] Perez-Riverol Y et al. 2016 Ten simple rules for taking advantage of Git and GitHub. PLoS Comput Biol. 12: e1004947 (10.1371/journal.pcbi.1004947) 27415786 PMC4945047

[59] Nielsen M 2012 Reinventing discovery: the new era of networked science. Princeton University Press See https://market.android.com/details?id=book-afqfFW8WV9cC.

[60] Lerner J, Tirole J 2003 Some simple economics of open source. J. Ind. Econ. 50, 197–234. (10.1111/1467-6451.00174)

[61] Fang Y, Neufeld D 2009 Understanding sustained participation in open source software projects. J. Manage. Inf. Syst. 25, 9–50. (10.2753/MIS0742-1222250401)

[62] Wicks SR, Rankin CH 1995 Integration of mechanosensory stimuli in *Caenorhabditis elegans*. J. Neurosci. 15, 2434–2444. (10.1523/JNEUROSCI.15-03-02434.1995) 7891178 PMC6578104

[63] Wicks SR, Roehrig CJ, Rankin CH 1996 A dynamic network simulation of the nematode tap withdrawal circuit: predictions concerning synaptic function using behavioral criteria. J. Neurosci. 16, 4017–4031. (10.1523/JNEUROSCI.16-12-04017.1996) 8656295 PMC6578605

